# Polyarginine Decorated Polydopamine Nanoparticles With Antimicrobial Properties for Functionalization of Hydrogels

**DOI:** 10.3389/fbioe.2020.00982

**Published:** 2020-08-18

**Authors:** Céline Muller, Emine Berber, Gaetan Lutzweiler, Ovidiu Ersen, Mounib Bahri, Philippe Lavalle, Vincent Ball, Nihal E. Vrana, Julien Barthes

**Affiliations:** ^1^Institut National de la Santé et de la Recherche Médicale, INSERM UMR 1121 “Biomaterials and Bioengineering”, Strasbourg, France; ^2^Université de Strasbourg, CNRS, Institut Charles Sadron, Strasbourg, France; ^3^IPCMS, Institut de Physique et de Chimie des Matériaux de Strasbourg, CNRS-UMRS 7504, Strasbourg, France; ^4^Université de Strasbourg, Faculté de Chirurgie Dentaire, Strasbourg, France; ^5^Spartha Medical, Strasbourg, France

**Keywords:** nanoparticles, polydopamine, polyarginine, hydrogel, antimicrobial properties

## Abstract

Polydopamine (PDA) nanoparticles are versatile structures that can be stabilized with proteins. In this study, we have demonstrated the feasibility of developing PDA/polypeptides complexes in the shape of nanoparticles. The polypeptide can also render the nanoparticle functional. Herein, we have developed antimicrobial nanoparticles with a narrow size distribution by decorating the polydopamine particles with a chain-length controlled antimicrobial agent Polyarginine (PAR). The obtained particles were 3.9 ± 1.7 nm in diameter and were not cytotoxic at 1:20 dilution and above. PAR-decorated nanoparticles have exhibited a strong antimicrobial activity against *S. aureus*, one of the most common pathogen involved in implant infections. The minimum inhibitory concentration is 5 times less than the cytotoxicity levels. Then, PAR-decorated nanoparticles have been incorporated into gelatin hydrogels used as a model of tissue engineering scaffolds. These nanoparticles have given hydrogels strong antimicrobial properties without affecting their stability and biocompatibility while improving their mechanical properties (modulus of increased storage). Decorated polydopamine nanoparticles can be a versatile tool for the functionalization of hydrogels in regenerative medicine applications by providing bioactive properties.

## Introduction

Tissue engineering and regenerative medicine use biomaterials, cells and bioactive agents to engineer implantable structures to replace damaged tissues and organs. In many cases, the size of the tissue to be replaced is considerable and once in the body, the engineered tissue might have immune privileged zones separated from the host circulation, which can create local havens for bacterial attachment and biofilm formation. This constitutes a situation similar to that of peri-implantitis (infections around dental implants) which can affect 5 to more than 10% of dental implants (Koldsland et al., 2010). As tissue engineering solutions become more and more common, there is a need to include antimicrobial agents as a safety precaution. However, such antimicrobial agents should not trigger the development of antimicrobial resistance or any undesired side effects and preferably they should have additional properties that can support the main function of the scaffold. One of the potential ways of achieving such an effect is the loading of nanoparticles functionalized with antimicrobial agents in scaffolds.

Polydopamine (PDA) films are versatile coatings obtained through the oxidation of dopamine (Lee et al., 2007) or other catecholamines (Kang et al., 2009) using either dissolved oxygen in basic solutions or strong oxidants (Wei et al., 2010; Ponzio et al., 2016). Knowing that PDA displays many structural similarities with eumelanin (Meredith and Sarna, 2006), the black–brown pigment of the skin, and that eumelanin grains display a structural hierarchy (Clancy and Simon, 2001), motivated us to explore specific controlled ways to synthesize PDA to obtain well defined and stable nanoparticles. Since eumelanin grains of the skin are always surrounded by proteins (Mani et al., 2001), the natural way to control the size of PDA particles is to add proteins (Chassepot and Ball, 2014) or other polymers (Arzillo et al., 2012; Mateescu et al., 2016) to the dopamine solution during its oxidation. The properties of transferrin capped PDA nanoparticles (Hauser et al., 2018) and the photothermal properties of such nanoparticles (Han et al., 2016) have already been explored. These inherent properties in term of photothermal sensitivity and versatile surface chemistry have shown great potential for biomedical applications. For example, PDA NPs photothermal properties have been used recently to develop drug release carriers for cancer therapy (Poinard et al., 2018; Wang et al., 2019). Moreover, the versatile surface chemistry of PDA NPs have allowed the conjugation of various bioactive agents (proteins, polyelectrolytes) though different mechanisms such as π-π stacking, electrostatic interactions and nucleophile addition of amine to quinone (Michael addition) (Ball, 2018). The possible functionalization of the PDA nanomaterials combined with their low toxicity made them good candidates for developing bioactive carriers with properties relevant for biomedical purposes such as anti-cancer, pro-angiogenic, anti-inflammatory, or antimicrobial activities (Ho and Ding, 2013; Chen et al., 2015; Li et al., 2019).

We recently demonstrated that chain-length controlled polyarginine (PAR) can be used as an antimicrobial agent inside thin films without having any adverse effect on mammalian cell behavior at concentrations several times higher than its minimal inhibitory concentration (Mutschler et al., 2016; Knopf-Marques et al., 2019). The polycationic nature of polyarginine is the underlying reason for such antimicrobial activity, as evidenced by the high frequency of arginine residues in natural antimicrobial peptides. Moreover, polyarginine has been shown to have cell penetration properties and has been utilized for DNA and RNA delivery (Kim et al., 2010; Hu et al., 2019). Thus, incorporating polyarginine into tissue engineered scaffolds such as hydrogels would not only provide the required antimicrobial properties, but it could also be used for the delivery of genes within the scaffold to enhance their bioactive properties. Therefore, PAR can potentially be used for the decoration of PDA nanoparticles while making them antimicrobial and ensuring that the decoration does not interfere with its antimicrobial activity.

Hydrogels due to their intrinsic properties in term of high-water absorption, viscoelastic properties and biocompatibility can closely simulate properties of living tissues. In particular hydrogels made of natural polymer-based components such as collagen, gelatin, chitosan or alginate are of particular interest since they can mimic the natural microenvironment of an extracellular matrix (Lee and Mooney, 2001; Hoffman, 2012). For this reason, these materials represent good candidates for biomedical applications such as drug delivery, wound dressing or tissue engineering (Hunt et al., 2014; Zilberman, 2015; Li and Mooney, 2016; Hamedi et al., 2018; Mohamad et al., 2019). Nevertheless, their use is still limited by their poor mechanical properties and their lack of stability in physiological condition. To overcome these limitations, multiple strategies have been proposed to reinforce hydrogels. These strategies include (i) the crosslinking of the hydrogel either chemically (creation of covalent bonds between macromolecular chains), physically (entanglement, ionic bonds, H-bonds) or biologically (nucleotide pairing, self-assembly, enzymatic crosslinking) (Place et al., 2009) or (ii) the use of fillers such as nanoparticles or nanofibers (Zhou and Wu, 2011; Sahraro et al., 2018; Sheffield et al., 2018). With both strategies the mechanical behavior of the hydrogel could be improved but with the use of fillers, the hydrogel’s bio-functionality can also be tuned by the intrinsic properties of nanoparticles (such as silver NP which are antimicrobial) (Ribeiro et al., 2017) or by functionalizing these nanoparticles with bioactive molecules. Thus, in this context, PAR decorated PDA particles can be used to render hydrogels antimicrobial and change their mechanical properties simultaneously.

The risk of contamination is a serious problem in implantable devices as infection can even lead to implant failure and in this aspect, most of the hydrogels used in the biomedical field are also concerned by this issue (Salomé Veiga and Schneider, 2013). As a consequence, antimicrobial strategies must be envisioned in all biomedical products development (Yu et al., 2007; Ng et al., 2014; Ribeiro et al., 2017). PDA-based nanoparticles have been previously utilized to elaborate, biocatalysts (El Yakhlifi et al., 2018), cell-targeting agents (Li et al., 2017), or theranostic agent (Dong et al., 2016) but to our knowledge, protein capped PDA nanoparticles have never been used for antimicrobial applications particularly in conjugation with hydrogels. Hence, we aim to develop a gelatin-based hydrogel with antimicrobial properties and enhanced mechanical properties through the loading of polydopamine nanoparticles decorated with an antimicrobial agent, polyarginine. This gelatin-based hydrogel should provide the optimal microenvironment for cell encapsulation while PAR decorated PDA particles will prevent bacterial contamination after implantation. To achieve this, the following hypotheses must be validated:

(i) PDA NPs should demonstrate the feasibility of being decorated with PAR,
(ii) The immobilization of PAR at the surface of PDA NPs should not interfere with antimicrobial properties of PAR,
(iii) PAR decorated PDA particles should retain their antimicrobial properties in gelatin hydrogels without compromising the biocompatibility of the hydrogels.

## Materials and Methods

### Materials

Dopamine hydrochloride (Mw = 189,64 Da, H8502, CAS: 62-31-7), Tris(hydroxymethyl) aminomethane (Mw = 121.1 Da, T-1503, CAS: 77-86-1) and Gelatin Type A from porcine skin (Mw = 5–10 × 10^4^Da) were purchased from Sigma-Aldrich (St Quentin Fallavier, France). Poly(L-arginine hydrochloride) with 30 arginine residues (PAR_30_, Mw = 5.8 kDa, CAS: 26982-20-7) was purchased from Alamanda Polymers (US). Microbial-Transglutaminase (Activa, 86-135 units/g) was kindly provided by Ajinomoto (Japan).

### Methods

#### Synthesis of Polydopamine Nanoparticles (PDA NPs) and PDA NPs Decorated With PAR_30_ (PDA-PAR_30_)

**FIGURE 1:**
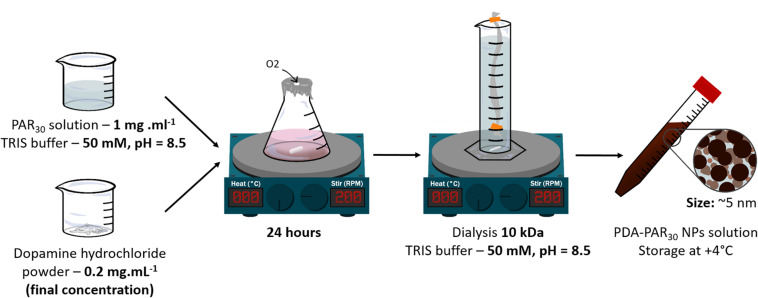
Different steps to synthesize PDAPAR_30_ NPs.

PAR_30_ powder was dissolved in TRIS buffer 50 mM at pH = 8.5 at a concentration of 1 mg.mL^–1^. Then the PAR_30_ solution was mixed with different masses of dopamine **hydrochloride** powder in order to obtain a final concentration of dopamine hydrochloride in solution ranging from of 0.2 to 0.5 mg.mL^–1^. The different formulations were noted respectively **PDA-PAR_30_ 0.2**, **PDA-PAR_30_ 0.3** and **PDA-PAR_30_ 0.5**. These solutions were stirred for 24 h under stirring (300 RPM) (Figure 1). After that, 7 consecutive steps of dialysis in 900 mL TRIS buffer were performed under stirring at + 4°C in order to remove the PAR30 non-deposited onto PDA NPs. We used dialysis cassette with a cut-off of 10 kDa which contained 12 mL of our NPs solution. Fresh buffer was added for each new dialysis step. Final solutions were stored at + 4°C. PDA NPs were synthesized using the same protocol just by removing PAR_30_ deposition step.

#### Synthesis Fluorescently Labeled PAR_30_-FITC NPs

PAR30 chains were labeled with fluorescein isothiocyanate (FITC, Sigma-Aldrich, France) using a protocol previously described by Mutschler et al. (2016). Briefly, for labeling PAR chains, PAR (15 mg.mL^–1^ in 100 mM NaHCO_3_ pH 8.3 buffer at pH = 8.3) was incubated with fluorescein isothiocyanate (FITC, Sigma-Aldrich, France) at a 1:2 molar ratio of PAR/FITC, at room temperature for 3 h. This solution was dialyzed against 1 L of water at 4°C with a Slide-A-Lyser Dialysis Cassette (Thermo Fischer Scientific Inc., United States), molecular weight cut off = 3500 g.mol^1^. PAR-FITC was then produced and stored in aliquots of 2 mL (0.5 mg⋅mL^–1^ in NaCl-TRIS buffer).

#### Dynamic Light Scattering and Zeta Potential Measurements

The hydrodynamic diameter of the PDA-PAR_30_ particles was measured by dynamic light scattering (Malvern Nano ZS) in a backscattering configuration (θ = 173°) and at a wavelength of 632.8 nm (HeNe laser). The intensity autocorrelation function obtained from a 1 mL suspension (after dialysis) was analyzed with the Contin algorithm. The volume distribution as a function of the hydrodynamic diameter was obtained thereof using a refractive index of 1.73 ± 0.02i at the wavelength of the experiment. At least three measurements were realized for each sample.

Zeta potential measurements were performed using Nano-ZS device (Malvern Instruments, United Kingdom). At least three measurements were realized for each sample in order to compare zeta potential between PDA NPs and PDA-PAR_30_ NPs.

#### Elaboration of Gelatin Hydrogel Loaded With PDA-PAR_30_ NPs (Gel-PDA-PAR_30_)

Gelatin hydrogel was prepared with gelatin type A at 6% w/v in PBS as a control (Gel-PBS). As PDA-PAR_30_ NPs were prepared in Tris buffer, Gel-PDA-PAR_30_ composite hydrogel was prepared in a PBS/Tris mixture at different ratios. For example, Gel-PDA-PAR_30_ was prepared by adding directly PDA-PAR_30_ NPs in Tris buffer solution to gelatin type A powder. In this condition PDA-PAR_30_ NPs were not diluted in the hydrogel. Then Gel-PDA-PAR_30_ 1:2 was prepared by first diluting PDA-PAR_30_ NPs in Tris buffer solution with PBS at a ratio 1:1 (Tris/PBS) (V/V) and then dissolving gelatin powder in this solution. The same method was used to prepare the other formulations. Then Gelatin solution was heated at + 40–50°C until complete solubilization. After that, microbial- Transglutaminase (TGA, enzymatic crosslinker) prepared in PBS at 20% w/v was added in a ratio 1–5 (200 μL of TGA for 1000 μL of gelatin). After a mixing step, 200 μL gelatin solution were poured into molds or 48 wells-plate. This crosslinking step was performed overnight at + 4°C. Finally, hydrogels were treated with UV light for 15 min before being stored at 4°C prior to use.

#### Microscopy Analyses

##### Scanning electron microscope (SEM)

NPs or Gel-NPs samples were let to dry overnight under a fume hood prior to be observed with a SEM Hitachi SU8010 microscope under 1 KeV voltage acceleration. Low-angle Back-scattered electron (LA-BSE) detector were used to acquire information both on the composition of the sample and on the topography.

##### Cryo-transmission electron microscope

For the cryo-TEM characterization, a drop of the same PDA-PAR_30_ particle suspension was deposited on a carbon coated electron transmission microscopy grid. The drop was aspired with filter paper to obtain a particle containing film covering the whole carbon membrane. Those coated grids were immersed in ethane at the temperature of liquid nitrogen, transferred on the cryo-holder and inserted in the electron microscope. Analysis was performed at 200 kV with a Jeol 2100F transmission electron microscope. To reduce the irradiation damage, the images were acquired using a low-density electron beam.

##### Epifluorescent microscope

Fluorescence pictures were acquired with the Nikon Eclipse Ti-S microscope with x10 objective equipped with Nikon Digital camera (DS-Q11MC, NIS-Elements software, Nikon, France) and processed with ImageJ^1^.

#### Rheology

Rheological properties were measured using a Kinexus Ultra + rheometer (Malvern Panalytical, United Kingdom). For the determination of the shear viscosity and viscoelastic properties (G’), we used a plate geometry of 20 mm diameter, a gap of 0.5 mm and the temperature was kept constant at 37°C. Shear viscosity measurement were carried out with a shear rate ranging from 10^–1^ to 10^3^ s^–1^. Gelatin and Gel-PDA-PAR_30_ 1:1 (non-diluted) were prepared as previously described without microbial-TGA and then 200 μL of these solutions were put in the rheometer at 37°C. Viscoelastic properties measurements were then performed with a frequency ranging from 0.1 to 50 Hz at a fixed strain of 0.5%. For these experiments, microbial-Transglutaminase was added to Gelatin and Gel-PDA-PAR_30_ solutions at a ratio 1:5 and then 200 μL of these solutions were put in the rheometer at 37°C and let crosslinked overnight before performing the sweep frequency experiment.

#### Stability (Enzymatic, pH 5, and PBS)

To study stability of our gelatin hydrogel containing PDA-PAR_30_ NPs (Gel-PDA-PAR_30_ 1:1) undiluted we used three different test configurations: (i) stability at physiological pH, (ii) in acidic condition, and (iii) in the presence of enzyme (collagenase).

For the experiment at physiological pH, we used PBS solution. Hydrogel samples were placed in 24 wells-plate with 1 mL of PBS 1X from Dutscher, at + 37°C. Measurements were carried out after vacuum-drying step for 4 h then we weighed samples. Finally, we put them in a new PBS solution and put them back at + 37°C until next measurement. We performed these measurements at different time points: initial measurement (before stability experiment) then on days 1, 4, 7 and 15. The following formula was used:

Remaining mass = W_t_/W_0_^∗^100 with W_t_ = weight of the hydrogel at time of measurement (t) and W_0_ = initial weight of the hydrogel.

For the experiment in acidic condition (pH = 5), protocol was the same as the previous one with the same follow-up at different time points. We used a citrate-phosphate buffer (0.15M, pH = 5).

To test enzymatic stability, we used collagenase from Clostridium histolyticum (Sigma-Aldrich) solution at 0.1% w/v. Measurements were performed using the same protocol but with different time points. Measurements were realized at t0, 30 min, 1 h and 2 h. All samples were washed three times with PBS 1X in order to remove collagenase enzyme, dried with paper and stock until next day to perform vacuum-drying and weigh. All results were compared to gelatin hydrogel without NPs condition. All samples were performed in triplicates.

#### Swelling

To study swelling ratio of our gelatin hydrogel containing PDA-PAR_30_ NPs undiluted (Gel-PDA-PAR_30_), we used the following protocol. We performed a first vacuum-dry step to obtain initial weigh. Then, samples were immersed in PBS 1X at + 37°C. Measurements were performed after drying briefly with a paper. We realized these measurements at different time points: 5, 10, 20, 30, 60, 120, 180, and 240 min. The following formula was used:

SW(%)=(W-tW)0/W1000*

with W_t_ = weight of the hydrogel at time of measurement (t) and W_0_ = initial weight of the hydrogel.

All results were compared to gelatin hydrogel without NPs condition. All samples were performed in triplicates.

#### Release Experiments

Release experiments of PDA-PAR_30_ NPs from Gelatin hydrogel were performed using Fluorescently labeled PAR_30_-FITC NPs as described in section “Methods.” Gelatin hydrogels composite with fluorescently labeled PDA-PAR_30_ NPs were produced as described in section “Elaboration of Gelatin Hydrogel Loaded With PDA-PAR30 NPs (Gel-PDA-PAR30)” (Gel-PDA-PAR_30_-FITC). The release experiments were performed with a 200 μL hydrogel in a 48 well plate at 37°C in a PBS solution (1 mL). Experiments were conducted with four different samples for 14 days. Supernatants were analyzed with a spectrofluorimeter (SAFAS Genius XC, Monaco). New PBS solutions (1 mL) were added after each analysis. For PAR_30_-FIT, the wavelength parameters were λex/λem = 495 nm/520 nm.

We are aware of the photosensitivity of FITC labeled molecules. Hence, we cannot exclude that during the release experiments some FITC molecules could be bleached, even if we used the lowest possible laser irradiance compatible with optimal detection efficiency. But if photobleaching would occur this means that we systematically underestimate the actual concentration of released FITC labeled particles, and hence our estimation of the release kinetics underestimates the real particle release.

##### Cytotoxicity assay

Cytotoxicity test was adapted from ISO STANDARD procedure 10993-5. Balb 3T3 clone A31 cell line (mouse fibroblast) was purchased from ATCC (CCL-163). The culture medium used was DMEM High Glucose with stable glutamine with pyruvate sodium (L0103-500) supplemented with 10% v/v of Fetal Bovine Serum (S1810-500) from Dutscher and 1% v/v penicillin-streptomycin (15140-122) from Gibco. Preculture was performed in 24 wells-plate by seeding 60,000 cells/well plate in order to obtain 80% confluency after 24 h. Two different cytotoxicity tests were performed for our samples. First, PDA-PAR_30_ NPs were added in solution at different dilutions onto cell layer (direct assay) for 24 h. We realized a PDA-PAR_30_ dilution of 1:10 in cell culture medium (ratio 1–9 Tris/Cell culture medium) to keep a normal cells viability, so the minimal dilution tested is 1:10. Then to increase the dilution of PDA-PAR_30_ NPs, we first diluted in Tris Buffer and then keep the same ratio of 1–9 Tris/Cell culture medium. Secondly, we tested gelatin hydrogels with PDA-PAR_30_ NPs at different dilutions, they were incubated in cell medium for 24 h in order to test release from hydrogels, in parallel of cells preculture. Gel-PDA-PAR_30_ were prepared as previously explained. These vehicles extracts were added onto cells layer (extract assay) for 24 h. All volumes were fixed at 1 mL. After exposure of samples onto cell layer (extract and direct assays) we performed MTT test. We also took bright field pictures at x10 before and after samples exposition to observe cells morphology. We compared all results to a growth control condition (cells cultured in cell culture medium without contact with hydrogel or NPs) defined as 100% viability. We also tested Tris buffer (in solution or in the hydrogel) as a negative control to check its non-cytotoxicity. We used sodium azide 3M (in solution or loaded in the hydrogel) as positive control. All samples were performed in triplicates.

##### Antibacterial assay Staphylococcus aureus

strain was purchased from ATCC (25923). *S. aureus* was cultured at + 37°C in a Mueller Hinton Agar (BD) for 24 h. Then one colony was transferred to 10 mL of Mueller Hinton Broth (BD) for 24 h at + 37°C with agitation for preculture step. The absorbance was read at 620 nm and we adjusted DO value to 0.001 to perform our assay, corresponding to a density of 8.10^5^ CFU.mL^–1^. As cytotoxicity assay, we tested PDA-PAR_30_ NPs in solution and also Gel- PDA-PAR_30_ composite hydrogel. For solution part, we kept the same ratio (samples:bacteria) in medium as 1–9, final volume is 100 μL (10 μL of NPs for 90 μL of bacteria in medium). Samples with bacteria were incubated for 24 h at + 37°C with agitation. Final absorbance was read at 620 nm in order to quantify bacteria growth or inhibition. For gelatin hydrogels part, hydrogels (200 μL) were directly produced in 48 wells-plate and sterilized by UV light exposure. After pre-culture of S. aureus, we added 300 μL of suspension with OD = 0.001 then we incubated for 24 h at + 37°C with agitation. OD was read at 620 nm. We compared PDA NPs and PDA-PAR_30_ NPs. In solution, OD of samples (PDA and PDA-PAR_30_) with bacteria were subtracted to OD the same samples without bacteria to remove PDA absorption (brown solution). All results were compared to a normal growth condition named “Bacteria,” defined as 100% growth. We also tested Tris buffer (in solution or hydrogel) as a negative control. We used antibiotics (Cefotaxime 0.1 μg.mL^–1^ + Tetracycline 10 μg.mL^–1^) as positive control. We also used PAR_30_ (10 μg.mL^–1^) without PDA as a positive control. All samples were performed in triplicates.

##### Bacteria live dead assay

We performed an additional test after antibacterial assay with gelatin hydrogels to evaluate the amount of bacteria alive or dead on the surface of samples. We used the BacLight Redox Sensor CTC Vitality Kit (Molecular Probes). SYTO 24 green-fluorescent nucleic acid staining was used at 0.001 mM to count all bacteria and CTC red staining was used at 50 mM to detect live bacteria.

## Results

### Synthesis and Characterization of PDA-PAR_30_ NPs

**FIGURE 2:**
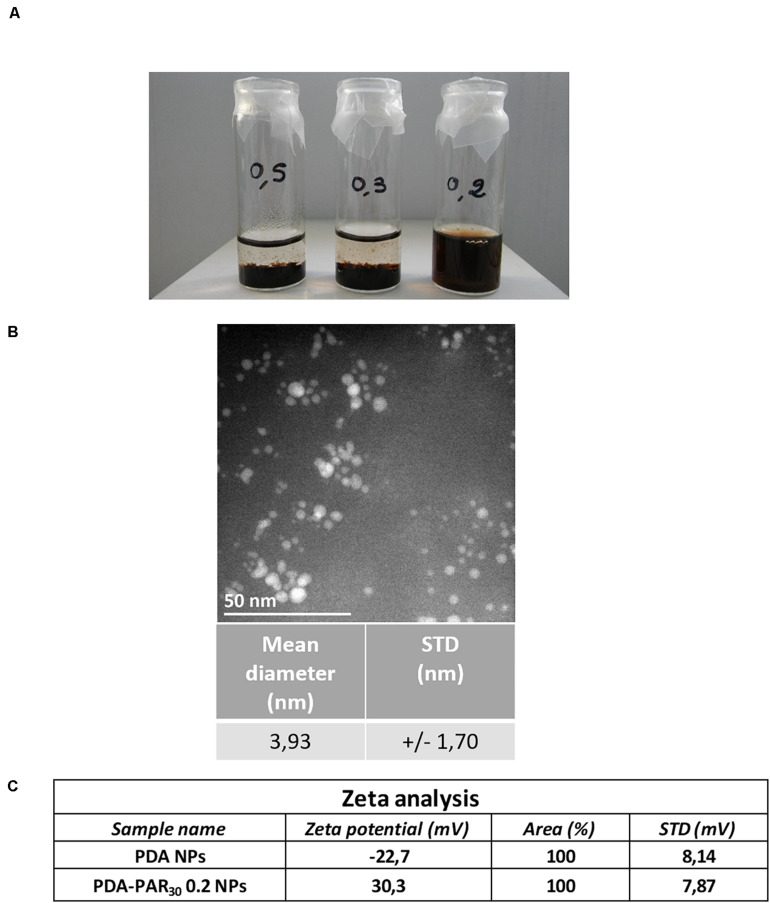
**(A)** Picture showing the stability after 2 months at 4°C of the different PDA-PAR_30_ NPs formulations using different concentrations of dopamine hydrochloride (0.5, 0.3, and 0.2 mg.mL^–1^) with a defined concentration of polyarginine (PAR) of 1 mg.mL^–1^. **(B)** Typical image of PDA-PAR_30_ 0.2 NPs obtained with Transmission Electron Microscope (TEM) and the subsequent size quantification results. **(C)** Results of zeta potential measurements performed on PDA-PAR_30_ 0.2 NPs as compared to pure PDA particles.

In this study, polydopamine nanoparticles (PDA NPs) were synthesized using a protocol developed by Chassepot and Ball (2014) In this work, the ability of proteins (either negatively or positively charged) to stabilize and control the size of dopamine aggregates formed in dopamine solutions upon oxidation in order to get functional nanoparticles was demonstrated. In the current work, we have used a chain-length controlled polycation, polyarginine with 30 residues (PAR_30_), known to have a strong antimicrobial activity (Mutschler et al., 2016). Then we have synthesized PDA-PAR_30_ nanoparticles by putting a PAR_30_ solution (1 mg.mL^–1^) in contact with different concentrations of dopamine hydrochloride solution ranging from 0.2to 0.5 mg.mL^–1^ in alkaline Tris Buffer (pH = 8.5) for 24 h under stirring (Figure 1). We first studied the influence of the dopamine concentration on the nanoparticle stability. After 2 months in a closed vial, only the formulation synthesized with 0.2 mg.mL^–1^ of dopamine hydrochloride (PDA-PAR_30_ 0.2) was still stable without apparent sedimentation (Figure 2A). For the other formulations containing 0.5 and 0.3 mg.mL^–1^ of dopamine hydrochloride (noted as PDA-PAR_30_ 0.5 and PDA-PAR_30_ 0.3), total sedimentation was observed after few days. The size of the aggregates was analyzed using dynamic light scattering technique (Supplementary Figure S1). Aggregates at the microscale were found for both formulations. The size of these aggregates has been estimated at approximately 2 μm and 4 μm for the formulations PDA-PAR_30_ 0.5 and PDA-PAR_30_ 0.3 respectively. For the stable formulation, PDA-PAR_30_ 0.2, Transmission Electron Microscope (TEM) analysis was performed to determine the size of the resulting nanoparticles (Figure 2B). Nanoparticles with a narrow size distribution of 3.9 ± 1.7 nm (mean diameter based on the measurement of 300 nanoparticles) were obtained. As a control, we also synthesized PDA NPs using the same protocol but without any decoration but these particles were not stable and formed aggregates between 1 and 10 μm (Supplementary Figure S1). The only way to obtain stable PDA based NPs was to use polypeptides or proteins to stabilize them. Thus, for the rest of the experimental work, we used the stable nanoparticles obtained with a solution of 0.2 mg. mL^–1^ of dopamine hydrochloride (PDA-PAR_30_ 0.2 condition). To further demonstrate the decoration of polydopamine NPs with chain-length controlled polyarginine, zeta potential measurements were performed. It is known that polydopamine is negatively charged above pH 5. Indeed, the measurement on PDA NPs gave a zeta potential value of -22.7 mV (Figure 2C). By adding PAR_30_ to the PDA particles, we obtained a zeta potential value of + 30.3 mV. The surface charge of the NPs switched from negative to positive values as PAR_30_ is a polycation. This demonstrates that the polycation was successfully deposited on the surface of PDA NPs. In the next step, we investigated the antimicrobial properties of PDA-PAR30 0.2 NPs to check if the PAR chains retained their biological activity once immobilized on the surface of PDA NPs. Indeed, our previous studies have demonstrated the need for mobility of PAR molecules in coatings to exert their antimicrobial activity and this mobility in particles was not guaranteed (Mutschler et al., 2016).

**FIGURE 3:**
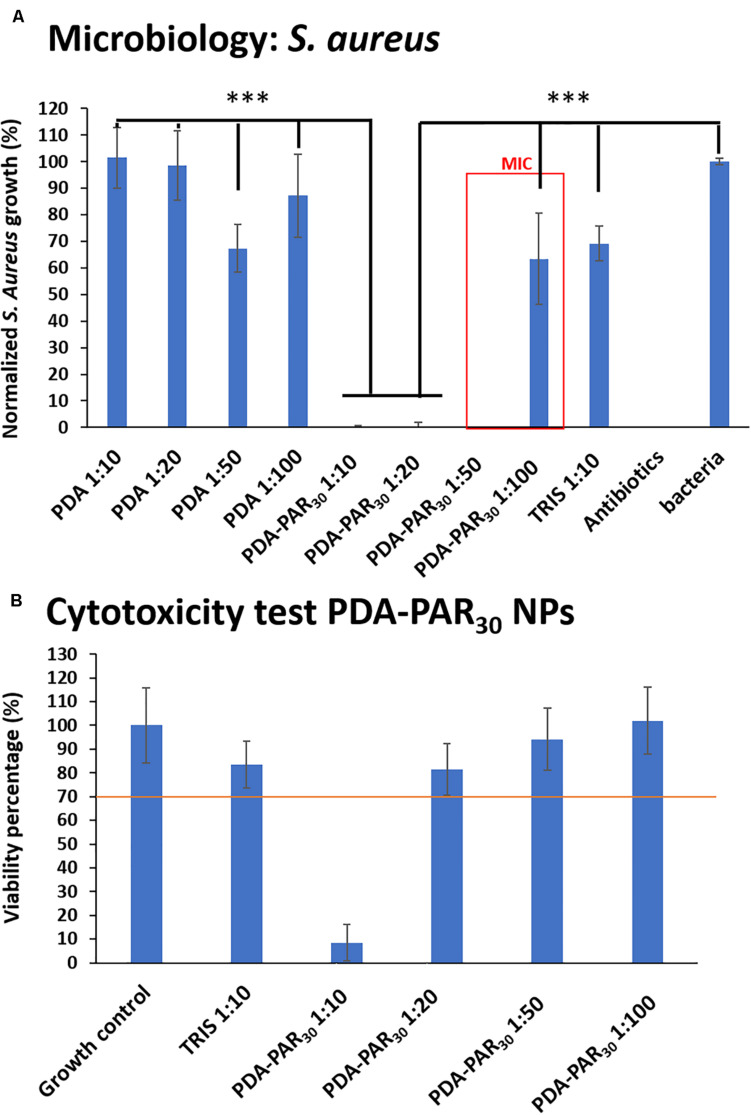
**(A)** Normalized S. aureus growth in supernatant after 24 h in contact with different PDA-PAR_30_ 0.2 NPs at different dilutions to determine the Minimum Inhibitory Concentration (MIC) (*n* = 3 and error bars correspond to standard deviations) (***p < 0.001). **(B)** Cytotoxicity test of the same NPs dilutions after 24 h of exposure with Balb 3T3 cells (*n* = 3 and error bars correspond to standard deviations).

We have tested different NPs dilutions ranging from 1:10 to 1:100 from the initial particle concentration and incubated them with *S. aureus* (8.10^5^ CFU.mL^–1^) for 24 h. As a control, we also synthesized unmodified PDA NPs and used them in the same range of dilution against *S. aureus*. The results are shown in Figure 3A. PDA-PAR30 0.2 NPs ranging from 1:10 to 1:50 in dilution completely inhibited bacterial growth (100% growth inhibition) then from the dilution 1:100, we were again able to observe a significant bacterial growth (40% growth inhibition for 1:100 dilution). Based on these results, we deduced that the MIC (minimum inhibitory concentration) of these PDA-PAR30 0.2 NPs was between 1:50 and 1:100. As it was difficult to estimate the true concentration of NPs, based on the absence of a precise knowledge about the dopamine oxidation-oligomerization reaction yield, we have performed this experiment on different batches of NPs coming from different syntheses done in identical conditions. The MIC was always estimated between 1:50 and 1:100 (Supplementary Figure S2) which means that the synthesis was reproducible, and we can assume that we always obtained comparable amount of NPs of similar size in each synthesis. As a control, the experiments carried out on PDA NPs without PAR30 decoration showed an absence of antimicrobial activity whatever the dilution used. This means that the antimicrobial activity of the NPs can be attributed to the presence of PAR_30_ and that PAR_30_ chains maintained their biological activity after immobilization on PDA NPs.

After the evidence of the antimicrobial properties of the NPs, it was necessary to check their cytotoxicity. For this purpose, we used Balb 3T3 cells as a well-established model (mouse fibroblasts). PDA-PAR_30_ 0.2 NPs at different dilutions in cell culture medium were put in contact with an almost confluent layer of 3T3 cells for 24 h and the viability of these cells was then estimated with a MTT assay and compared to a control (normal medium without NPs). A material is defined as cytotoxic if a decrease of at least 30% in viability compared to the control is observed according to ISO/EN 109935. The obtained results are presented in Figure 3B and images of the cell morphology are given in Supplementary Figure S3. As a control, we also investigated the cytotoxicity of Tris Buffer at a dilution 1:10 which represents the highest concentration of Tris used in the experiments to dilute NPs in the cell culture medium. At this concentration of Tris Buffer, it was shown that the buffer was not cytotoxic. This indicates that for the rest of the experiment, if a cytotoxic effect is monitored, it can only be attributed to the presence of PDA-PAR nanoparticles. Finally, it was demonstrated that for PDA-PAR_30_ 0.2 NPs, only the 1:10 dilution exhibited a cytotoxic behavior with a decrease in viability of about 90%. Hence, we were able to conclude that PDA-PAR_30_ 0.2 NPs were non-cytotoxic at dilutions higher than 1:10.

### Design and Characterization of Antimicrobial Gelatin Hydrogels Loaded With PDA-PAR_30_ NPs (Gel-PDA-PAR_30_)

**FIGURE 4:**
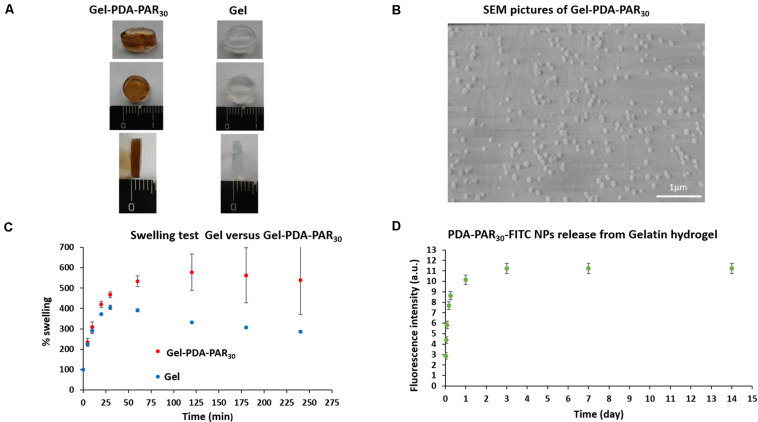
**(A)** Macroscopic pictures of the resulting Gelatin NPs hydrogel composite (Gel-PDA-PAR_30_) compared to pure Gelatin hydrogel (Gel). **(B)** Scanning Electron Microscopy images of the Gel-PDA-PAR_30_ hydrogel. **(C)** Swelling properties of the Gel-PDA-PAR_30_ hydrogel compared to pure Gelatin hydrogel at different time points in PBS at 37°C (n = 3 error bars correspond to standard deviations). **(D)** Kinetic of PDA-PAR_30_-FITC NPs (non-diluted) release from Gelatin hydrogel at 37°C in PBS for 15 days. Fresh supernatant was added after each recording to perform cumulative release over time (*n* = 3 error bars correspond to standard deviations).

We have previously demonstrated that decoration of PDA NPs with PAR_30_ confers antimicrobial properties. In the second part of the study, we have incorporated these PDA decorated NPs into gelatin hydrogels to develop an antimicrobial hydrogel that can be further used as an implant surface coating to fight infection after implantation while providing a degradable environment for remodeling. For this purpose, PDA-PAR_30_ 0.2 NPs were incorporated into gelatin type A hydrogel without any dilution (Gel-PDA-PAR_30_ and the hydrogels were then crosslinked with microbial-Transglutaminase in order to reinforce their mechanical properties and stability. This hydrogel Gel-PDA-PAR_30_ 1:1 is depicted in Figure 4A and compared to gelatin hydrogel without NPs (Gel). We observed a brown color for the hydrogel loaded with NPs which is the characteristic color of PDA. The presence of NPs at the surface of the gelatin hydrogel was checked using SEM and it was found that the particles were homogeneously distributed. However, we noticed that once incorporated in the hydrogel, there was an increase of particle size from few nanometers (as found for solution dispersed NPs, Figure 2B) to almost 100 nm. This can be explained by the aggregation of the NPs during the hydrogel formation (Figure 4B). We then investigated the swelling properties of the composite hydrogels and compared them to pure gelatin hydrogels (Figure 4C). It was found that the addition of NPs in the gelatin network had an effect on the swelling behavior: a significant increase with a value of 500% was monitored, compared to pure gelatin hydrogel where a value of only about 300% was measured. This could be explained by Donnan effect due to the high positive charge density of the PAR covered NPs inducing a strong incorporation of counteranions and hence of hydration water (Rathna et al., 1996). Then, the release of PDA-PAR_30_ 0.2 NPs from the gelatin hydrogel was studied using fluorescently labeled NPs with PAR_30_-FITC (Figure 4D). We observed a burst release of the NPs during the first hours with most of the NPs released during the first 3 days. However, the hydrogel was still brown after 3 days of release (data not shown), which means that some of the NPs were still present in the gelatin network even if no further release was recorded.

**FIGURE 5:**
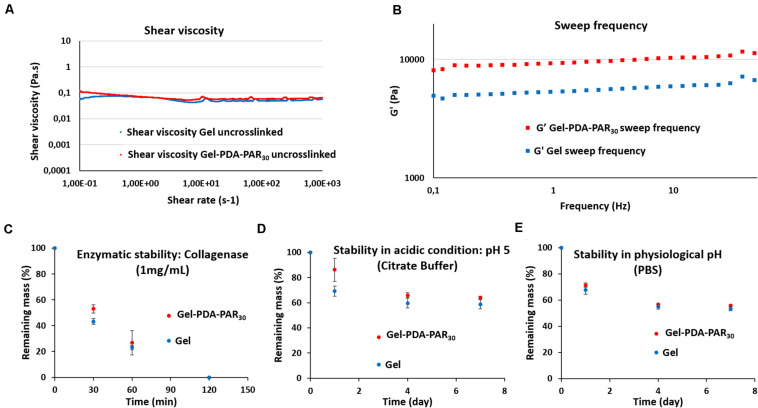
Rheological properties and stability of both gelatin (Gel) and Gelatin NPs composite (Gel-PDA-PAR_30_) hydrogels. **(A)** Shear viscosity as a function of shear rate for both Gel and Gel-PDA-PAR_30_. **(B)** Storage modulus (G’) as a function of frequency for both conditions tested (Gel vs. Gel-PDA-PAR_30_). Stability of the different hydrogels tested (Gel vs. Gel-PDA-PAR_30_) at 37°C in a relevant biological medium, **(C)** enzymatic stability in collagenase; **(D)** in acidic condition (pH = 5) and in **(E)** physiological pH (pH = 7.4). The results are expressed as a percentage of remaining mass at different time points (*n* = 3 error bars correspond to standard deviations).

Next, we investigated the rheological properties of the gelatin-PDA-PAR_30_ hydrogel composite to determine the influence of the addition of PDA-PAR_30_ NPs on the mechanical properties. We first analyzed the shear viscosity in solution of the two formulations (Gel and Gel-PDA-PAR_30_) without the crosslinking agent, microbial Transglutaminase (Figure 5A). The shear viscosity of both formulations remained constant over a large range of shear rates (from 0.1 to 1000 s^–1^). Moreover, the addition of nanoparticles in the formulation did not affect the shear viscosity since the values of both Gel and Gel-PDA-PAR_30_ formulations were similar with values ranging from 0.1 to 0.06 Pa.s depending on the shear rate applied. After demonstrating that the addition of NPs in the formulation did not affect their viscosity, we crosslinked both formulations with microbial-transglutaminase and studied the viscoelastic properties of the resulting hydrogels. The two hydrogels were crosslinked for at least 16 h *in situ* in the rheometer to ensure complete crosslinking of the network. Then we recorded the storage modulus of the two hydrogels as a function of frequency (Figure 5B). Over the frequency range tested (0.1–50 Hz), it was found that the storage modulus (G’) of the two formulations increased slightly as a function of frequency. In addition, we observed a higher storage modulus in the case of Gel-PDA-PAR_30_ hydrogel composite with values between 8 to almost 11 kPa, whereas for the gelatin hydrogel the values were between 5 and 7 kPa. Thus, the addition of NPs within PDA-PAR hydrogel enhanced its viscoelastic properties. We assumed that the NPs acted as a filler in the structure of the hydrogel.

After characterization of the mechanical properties of the hydrogels, we performed a stability study in different conditions (Figures 5C–E): enzymatic stability in the presence of collagenase, stability in acidic conditions (pH = 5) as a model of infection and stability at physiological pH in PBS. All these experiments were conducted at 37°C and the mass of hydrogels were recorded as a function of time. For enzymatic stability, hydrogels were incubated in 1 mL of collagenase solution (1 mg.mL^–1^) at 37°C. In Figure 5C, it is shown that the two formulations, Gel or Gel-PDA-PAR30, were completely degraded after 2 h with a slightly higher degradation rate for Gel condition. For the stability at pH = 5, hydrogels were incubated in 1 mL of citric buffer and experiments were performed for 7 days with an estimation of mass loss on days 1, 4, and 7 (Figure 5D). After the first day, the hydrogel containing PDA-PAR_30_ NPs showed better stability (86% of remaining hydrogel vs. 69% for the pure gelatin hydrogel). But during the next days, the degradation was indistinguishable for both formulations. After 7 days, the difference between the two hydrogels was not significant (64% degradation for Gel-PDA-PAR_30_ vs. 59% for Gel). The last stability experiment was also conducted for 7 days in PBS buffer at pH = 7.4 (Figure 5E) and the results showed the same degradation profile between the two formulations with values of remaining hydrogel after 7 days of about 55% (56% for Gel-PDA-PAR_30_ vs. 53% for Gel). With this set of experiments, we can conclude that the addition of PDA-PAR_30_ NPs in the gelatin structure only increased the viscoelastic properties of the hydrogel (increase of G’, Figure 5B) but did not change the stability of the resulting hydrogels compared to pure gelatin hydrogels.

**FIGURE 6:**
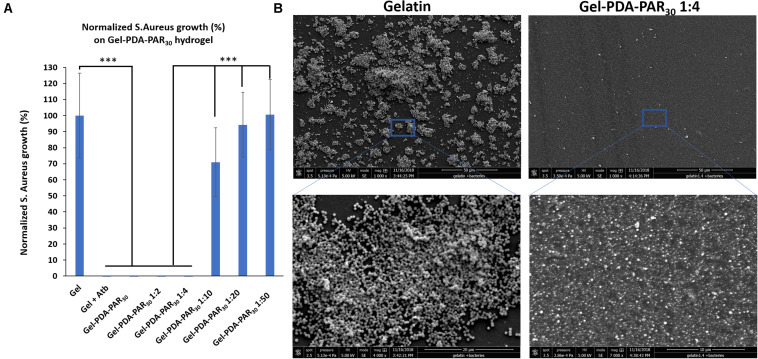
**(A)** Normalized *S. aureus* growth in supernatant after 24 h in contact with Gelatin NPs composite hydrogel loaded with NPs (Gel-PDA-PAR_30_) at different dilutions (*n* = 3 and error bars correspond to standard deviations) (***p < 0.001). **(B)** SEM images of Gelatin vs. Gel-PDA-PAR_30_ 1:4 (NPs diluted 4 times in the hydrogel matrix) after 24 h of exposition with S. aureus.

Once the hydrogel composites were characterized, their antimicrobial activity starting from a dilution 1:1 (no dilution) and until 1:50 was tested against *S. aureus* (Figure 6A). Hydrogels were prepared in PBS but as the NPs were prepared in Tris Buffer, the final hydrogel composite was prepared in a PBS/Tris mix. Gel-PDA-PAR30 hydrogels have been found to exhibit complete antimicrobial activity with 100% growth inhibition after 24 h in contact with S. aureus until a dilution of 1: 4. For higher dilutions (1:10 and 1:50), the antimicrobial activity was lost because the NPs were probably too diluted in the hydrogel to maintain biological activity. SEM analyses were also performed to compare the behavior of Gel-PDA-PAR30 (1: 4) vs. Gel (Figure 6B). After 24 h of contact with *S. aureus*, the bacteria fully colonized gelatin hydrogel and started to form a biofilm whereas with the NPs loaded hydrogel almost no bacteria were found. A zoom on the Gel-PDA-PAR30 surface made it possible to show the presence of particles on the surface of the hydrogel which confer antimicrobial activity.

**FIGURE 7:**
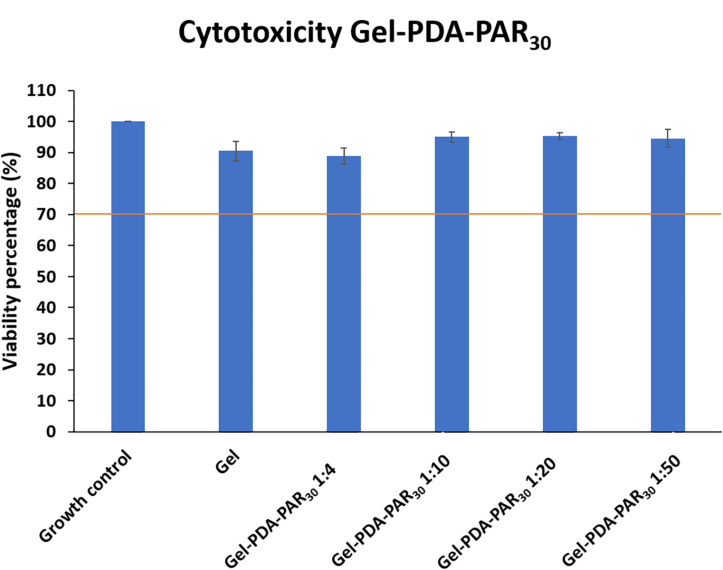
Cytotoxicity test with Balb 3T3 cells of Gelatin NPs composite hydrogel loaded with NPs (Gel-PDAPAR_30_) at different dilutions to determine the threshold of NPs toxicity while keeping the antimicrobial activity of the resulting hydrogel (*n* = 3 and error bars correspond to standard deviations).

These results were also in agreement with the metabolic activity of bacteria, using CTC/Syto 24 staining where no viable bacteria were found on the hydrogel loaded with NPs (Supplementary Figure S4). For final applications, the gel formulation with NPs diluted in a ratio 1:4 seemed to be optimal. Then, the cytotoxicity of the formulation Gel-PDA-PAR_30_. was tested with Balb 3T3 cells using extraction method following ISO Standard 10993-5 recommendations. As the hydrogel composite was prepared in a PBS/Tris mixture, we first tested the cytotoxicity of pure gelatin hydrogels prepared in either Tris or PBS and then the cytotoxicity of hydrogel prepared at different ratios of PBS/Tris (Supplementary Figure S5). It was found that none of the configurations tested exhibited cytotoxicity with viability up to 70% maintained in all cases. Then, we prepared Gel-PDA-PAR_30_ hydrogel composites starting from a dilution 1:4 and until 1:50 (NPs were also diluted from 1:4 to 1:50 in the hydrogel) and studied their cytotoxicity. None of the formulations tested were cytotoxic and a viability up to 85% was maintained (Figure 7). The resulting cell morphology is given in Supplementary Figure S6 and there was no evidence of anormal cell shape. The formulation 1:4 was tested toward Balb 3T3 cells and its viability was estimated to be around 90%. Hence we can conclude that this formulation was not cytotoxic. Thus, we have designed a powerful antimicrobial hydrogel only based on natural and bioderived materials, i.e., gelatin, polydopamine, and polyarginine. An adequate formulation was obtained both in terms of antimicrobial activity with 100% inhibition of bacterial growth and in terms of biocompatibility.

## Discussion

### Polyarginine Decorated PDA NPs Are Stable and Antimicrobial

Polydopamine NPs-based chemistry is versatile and should open the road to the development of bioactive biomaterials with the possibility of decorating nanoparticles with multiple bioactive agents allowing us to design hydrogel composites with bioactive properties. As these particles have a wide range of inherent properties as a potential substrate for photothermal effect or as quenchers and catalysts, they can be used in many biomedical applications including cancer therapy and stimuli responsive drug delivery. Adding a layer of bioactive agents on this structure creates a tool that is highly modular and can have application in tissue engineering and regenerative medicine for theranostic or biosensing. In this study, our main goal was to create stable polydopamine based nanoparticles with antimicrobial properties and to do that we have used a known polypeptide antibiotics (polyarginine).

We have first demonstrated that the addition of a sufficient amount of PAR_30_ to a sufficiently diluted solution of dopamine, 0.2 mg.mL^–1^, in Tris buffer allowed to produce small and stable PDA-PAR nanoparticles having an average diameter of 3.9 ± 1.7 nm and displaying a positive zeta potential of about + 30.3 mV compared to pristine PDA nanoparticles having a zeta potential close to -23 mV in the same conditions (Figure 2D). It is well known in colloid science that a high concentration of capping polymers leads to a fast adsorption on the forming particles and allows hence to reduce the occurrence of particle bridging and simultaneously a reduction in the average particle size. In our experiments the concentration of PAR30 was hold constant and the dopamine concentration (leading to the formation of PDA) was progressively decreased. We obtained stable particles for low dopamine concentrations, hence in the case where the molar fraction of the capping polymer, PAR30, was high (Butt et al., 2013).

Polyarginine, a positively charged polymer, has been originally used as a highly efficient cell penetrating peptide for gene delivery applications. Beyond this cell penetrating properties, due to its polycationic nature, polyarginine is a widely used component to build polyelectrolyte multilayers. Recently, it has also been shown that PAR_18_ and also its D-enantiomer have neuroprotective properties (Meloni et al., 2019). As mentioned before, polyarginine also has antimicrobial activity whose efficacy is related to the mobility of PAR chains within polyelectrolyte structures (Dong et al., 2016). In solution, a wide range of polyarginine chain lengths have shown antimicrobial activity, however, in film configurations where polyarginine was in interaction with polyanions (heparin, chondroitin sulfate, alginate, hyaluronic acid) only a narrow range of chain length and only films with HA were active. Thus, decoration of polyarginine on PDA particles might have hindered the antimicrobial activity of polyarginine due to the lack of mobility.

However, our experiments demonstrated that polyarginine chains on PDA surfaces maintain their antimicrobial capacity. Indeed, the PDA-PAR_30_ 0.2 NPs display an antimicrobial activity against *S. aureus* up to a dilution of about 1:50–1:100 (Figure 3A) whereas the PDA NPs are not antimicrobial whatever their dilution; this demonstrates that the antimicrobial activity arises from the presence of PAR on its surface and also that the particle formation does not hinder the antimicrobial activity of PAR, excepting the cases of thin layer-by-layer coatings where the antimicrobial properties depend on the polyanion. his is due to the fact that, even if they are immobilized, the PAR chains are on the surface of the particles, they are therefore free to interact electrostatically with the bacterial membranes even if the interaction is partially shared with the interaction with the surface of the PDA. Our experimental results clearly demonstrated that such interactions cannot overshadow the antimicrobial properties of polyarginine.

### PDA-PAR NPs Incorporated Hydrogels Have Improved Viscoelastic Properties and Antimicrobial Capacity

The need for antimicrobial coatings for non-degradable implants has been long recognized such with systems such as TYRX^TM^ antimicrobial, degradable polymer pouch used for pacemakers in clinical applications. The surface of non-degradable implants, can create an immune-privileged zone that can facilitate the bacterial attachment and the biofilm formation. For example, the rate of infections with fixation devices were estimated between 2 and 5% The replacement of passive implants with engineered artificial tissues can in one way alleviate this problem as the porous nature of such scaffolds will enable the angiogenesis. Consequently, the implanted structure will integrate with the host immune system rather than being an isolated surface. But, on the other hand, materials such as natural polymers (as for instance collagen, gelatin, hyaluronic acid etc., with certain exceptions like chitosan) used for scaffolds generation can also boost the attachment and proliferation of bacteria just as well as the host tissue. Moreover, potential use of engineered tissues in non-sterile wounds would also necessitate an antimicrobial component. Previously, such precautions has been tried to be put in place by either doping hydroxyapatite with silver or copper to confer antimicrobial activity to it (Sahithi et al., 2010; Stanić et al., 2011) or incorporation of silver based nanoparticles in the scaffold formulations (Saravanan et al., 2011). Specifically, with gelatin foams crosslinked with genipin, Yazdimamaghani et al. (2014) demonstrated antimicrobial activity against *E. coli* and *S. aureus* by *in situ* formed silver nanoparticles. However, it has been shown that silver nanoparticles are less cytotoxic compared to silver sulfadazine which at high concentrations can demonstrate hepatotoxicity in clinical settings (Trop et al., 2006). Moreover, at the cellular level, it has been shown that silver exhibits mild cytotoxicity against mesenchymal stem cells, which are one of the major cell sources in tissue engineering (Samberg et al., 2012). Thus, alternative antimicrobial solution would be beneficial, particularly in formats that would also contribute to the mechanical properties of the overall scaffold in tissue engineering. The nanoparticles developed in this study could potentially achieve these two effects concomitantly in the context of hydrogels.

In this study, to demonstrate such utility of PDA-PAR NPs, we have used gelatin hydrogels, which creates a highly amenable surface for bacterial attachment as demonstrated by our results. The incorporation of the particles in the gel structure resulted in an increase in G’ values which indicates a slightly stiffer hydrogel due to the nanoparticle based reinforcement. The incorporation of the particles in the gel structures could decrease their mobility and as a result their antimicrobial capacity, however, experiments showed that it was possible to maintain antimicrobial activity of the NPs at dilutions below the cytotoxic limits, the reason for this observation being the ability of the hydrogels to release the NPs as demonstrated by the release experiments. The relatively controlled manner of release of the NPs also points out using the hydrogels as a potential delivery system for such NPs. The comparison of Figure 4D (release experiment) and Figure 5E (stability of gelatin hydrogel composite in PBS) showed that 90% of the nanoparticles were released after 1 day of experiment while only 30% of the hydrogel was degraded in the same period which means that the release mechanism can be mainly attributed to the diffusion of NPs even if there is always a competition between passive release (diffusion) and degradation of the hydrogel.

## Conclusion

Herein, we demonstrated that PDA-based nanoparticles with a controlled size can be obtained in the presence of a polycationic antimicrobial peptide without interfering with the antimicrobial properties of the polypeptide. The system was effective not only in a stand-alone configuration but also within hydrogels. The incorporation of the resulting NPs in gelatin hydrogels as a model of tissue engineering scaffolds leads to hydrogels with antimicrobial capacity and improved mechanical properties. These NPs can be used to render tissue engineering scaffold antimicrobial; particularly in the cases where the slow kinetic in the cell growth can result in bacterial pouches while contributing to the mechanical properties of the scaffold. Last but not least, we showed that this hydrogel can release NPs which would also allow to use it for drug release applications.

## Data Availability Statement

All datasets presented in this study are included in the article/Supplementary Material.

## Author Contributions

CM, GL, EB, MB, and JB performed the experiments. JB, NV, and PL designed the experiments. JB, NV, VB, OE, and PL contributed to the interpretation of the results and the writing of the manuscript. All authors contributed to the article and approved the submitted version.

## Conflict of Interest

NV was employed by company Spartha Medical. The remaining authors declare that the research was conducted in the absence of any commercial or financial relationships that could be construed as a potential conflict of interest.

## Footnotes

1 https://imagej.nih.gov/ij/
