# Supplementary material for: Polyarginine Decorated Polydopamine Nanoparticles With Antimicrobial Properties for Functionalization of Hydrogels
Source: Front Bioeng Biotechnol. 2020 Aug 18;8:982. doi: 10.3389/fbioe.2020.00982 (PMC7461895; doi:10.3389/fbioe.2020.00982)
Supplement: Supplementary file 1 [file Data_Sheet_1.docx]

Supplementary Material

Polyarginine decorated Polydopamine nanoparticles with antimicrobial properties for functionalisation of hydrogels

**C. Muller^1^, E. Berber^1^, G. Lutzweiler^1,2^, O. Ersen^4^, M. Bahri^4^, P. Lavalle^1^, V. Ball^1, 3^, N.E. Vrana^1,5^, J. Barthes^1*^**

^1^ Institut national de la Santé et de la Recherche Médicale, INSERM UMR1121 “Biomaterials and Bioengineering”, 11 Rue Humann, 67085 Strasbourg, France.

^2^ Université de Strasbourg, CNRS, Institut Charles Sadron, 23 rue du Loess, 67034 Strasbourg, France.

^3^ Université de Strasbourg, Faculté de Chirurgie Dentaire, 8 rue Sainte Elisabeth, 67000 Strasbourg, France.

^4^ IPCMS, Institut de Physique et de Chimie des Matériaux de Strasbourg, CNRS-UMRS7504, 23 rue du Loess, 67034 Strasbourg, France.

^5^ Spartha Medical, 14B rue de la Canardière, 67100 Strasbourg, France.

*** Correspondence:**Corresponding Author: Julien Barthes
jbarthes25@gmail.com

*
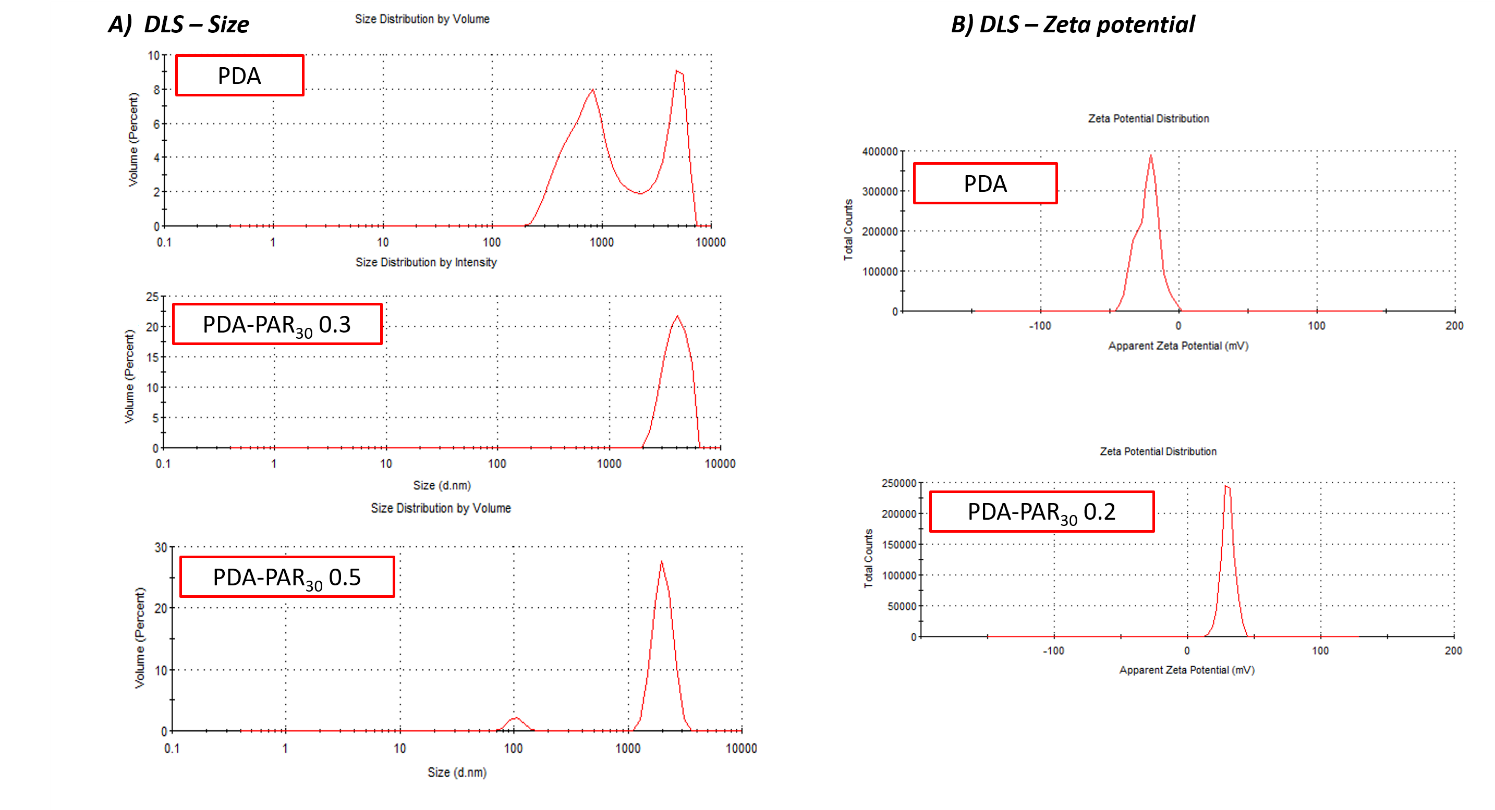
*

**Supplementary Figure 1**. A) Dynamic Light Scattering measurements (DLS) to determine particles size for the following particles formulations i) PDA, ii) PDAPAR_30_ 0.3 and ii) PDAPAR_30_ 0.5. B) Zeta potential measurements performed on PDA-PAR_30_ 0.2 NPs compared to pure PDA particles.


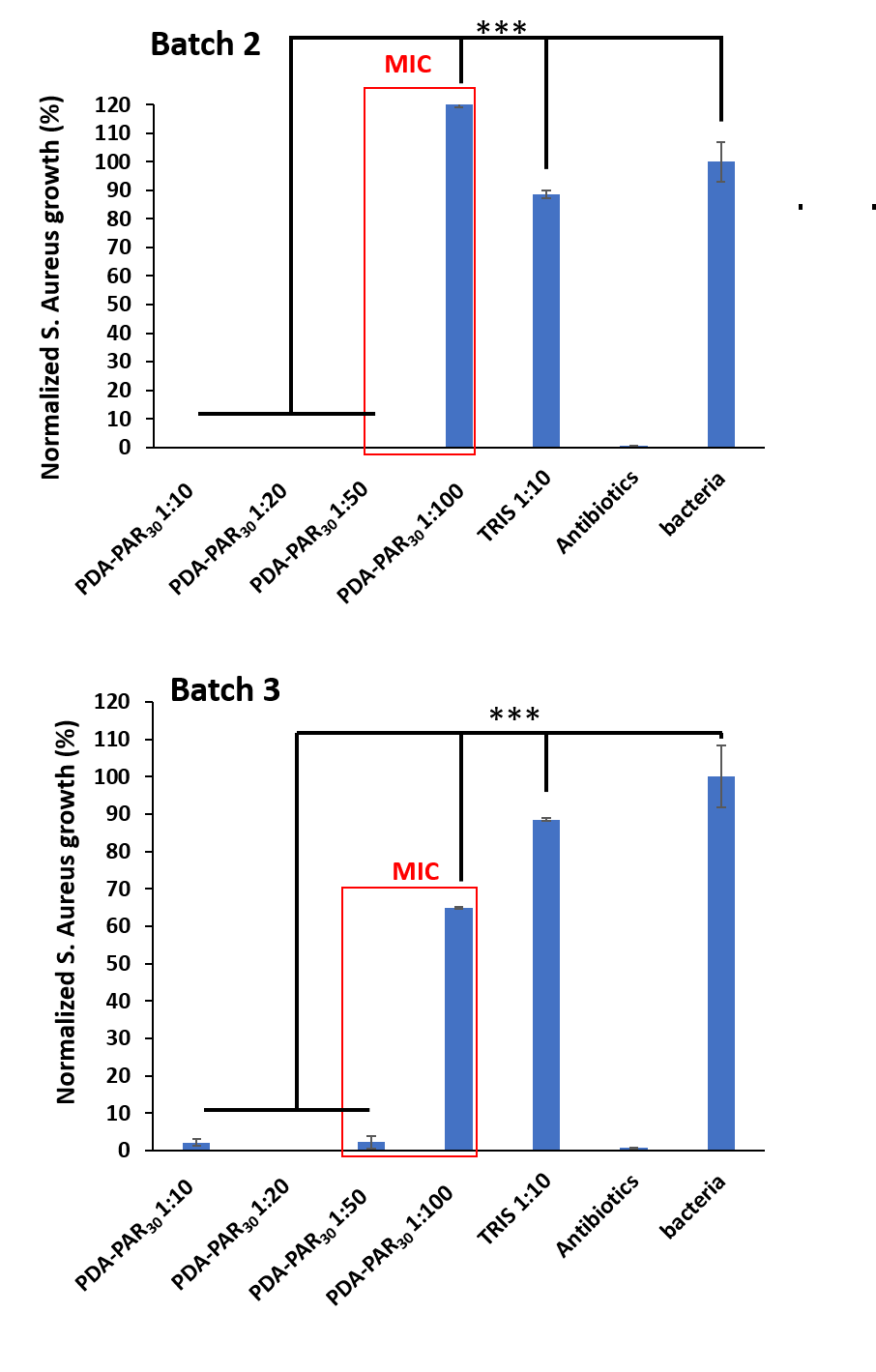


**Supplementary Figure 2**. Normalized S. aureus growth in supernatant after 24h in contact with different PDAPAR_30_ 0.2 NPs at different dilution to determine the Minimum Inhibitory Concentration (MIC). Experiments were performed with two different batches of NPs coming from different synthesis done in identical conditions (n=3 and error bars correspond to standard deviations) (***p < 0,001).


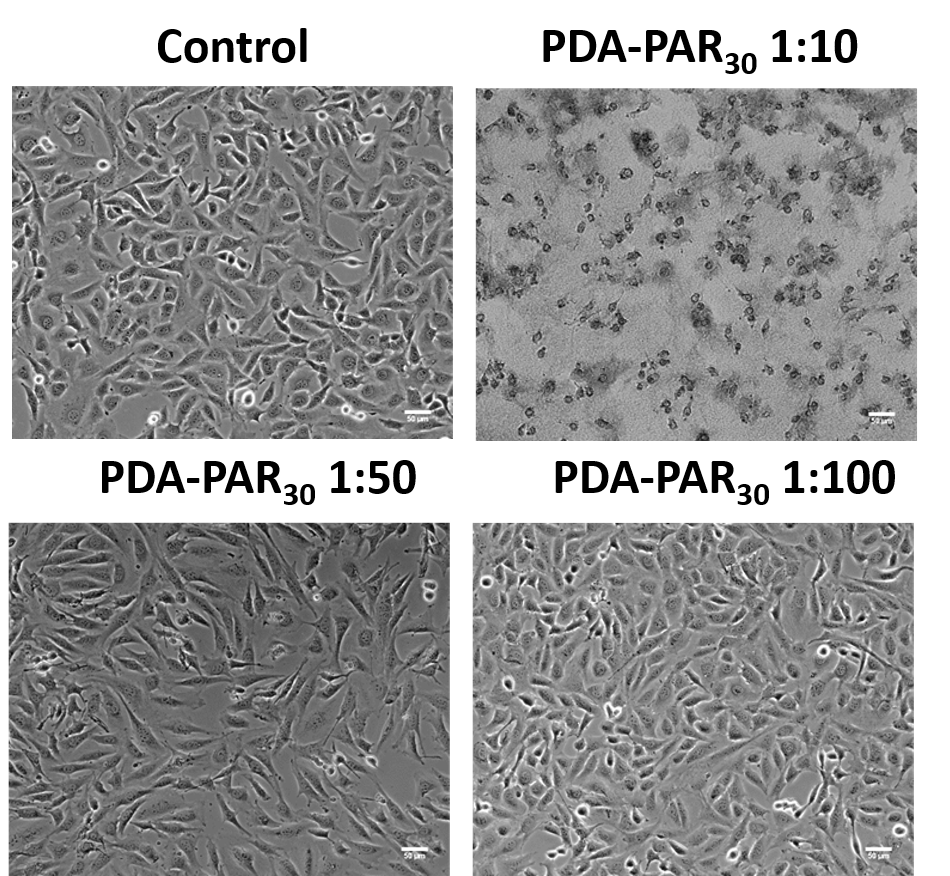


**Supplementary Figure 3**. Balb 3T3 cell morphology using bright field microscope after 24 hours of exposure with PDAPAR_30_ 0.2 NPs in solution at different dilutions (scale bar = 50µm).


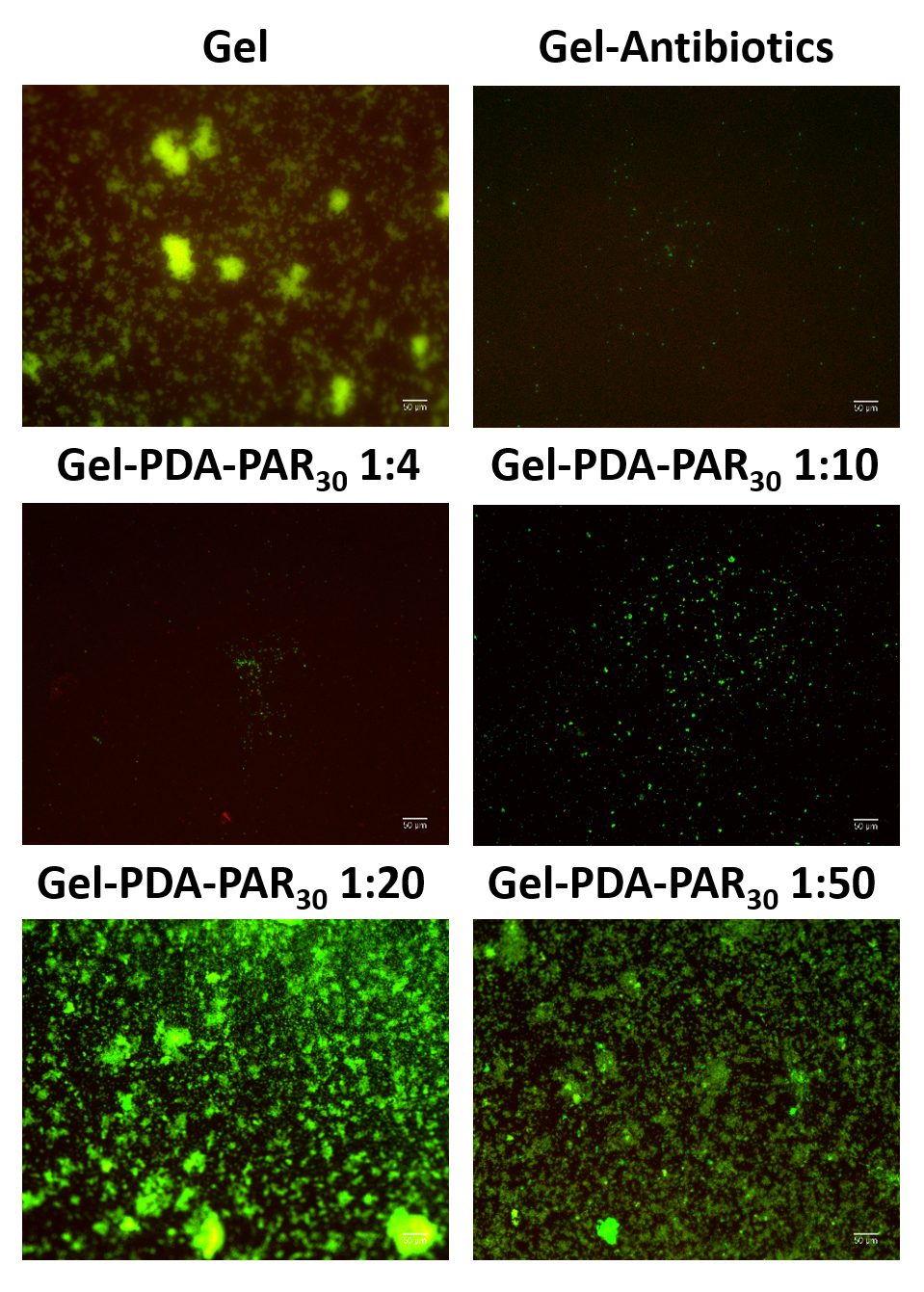


**Supplementary Figure 4**. Confocal images of S. aureus after 24h of contact with Gelatin NPs composite hydrogel loaded with NPs (PDAPAR_30_) at different dilutions (n=3 and error bars correspond to standard deviations). Confocal images in the green channel correspond to SYTO®24 labeling for counting all bacteria and in the red channel to healthy bacteria through metabolism of CTC (5-cyano- 2,3-ditolyl tetrazolium chloride), into an insoluble, red fluorescent formazan (scale bare = 50µm).


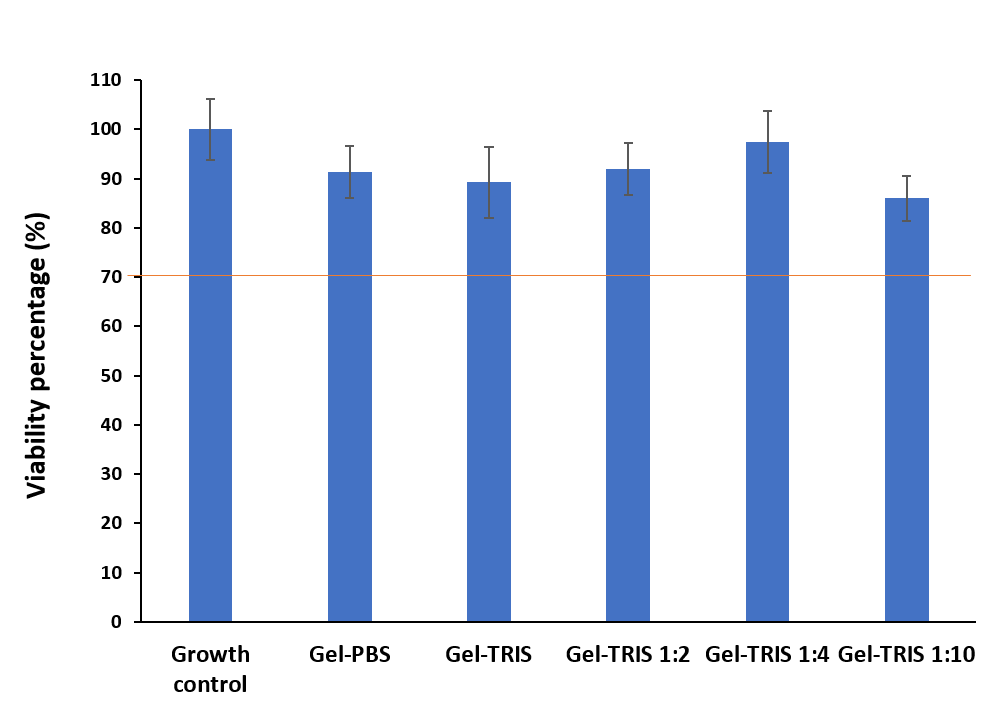


Supplementary Figure 5. Cytotoxicity test of gelatin hydrogels prepared in Tris buffer, PBS or in a mix PBS/Tris with Balb 3T3 cells to determine the potential toxicity of Tris buffer and the resulting gelatin hydrogel (n=3 and error bars correspond to standard deviations).


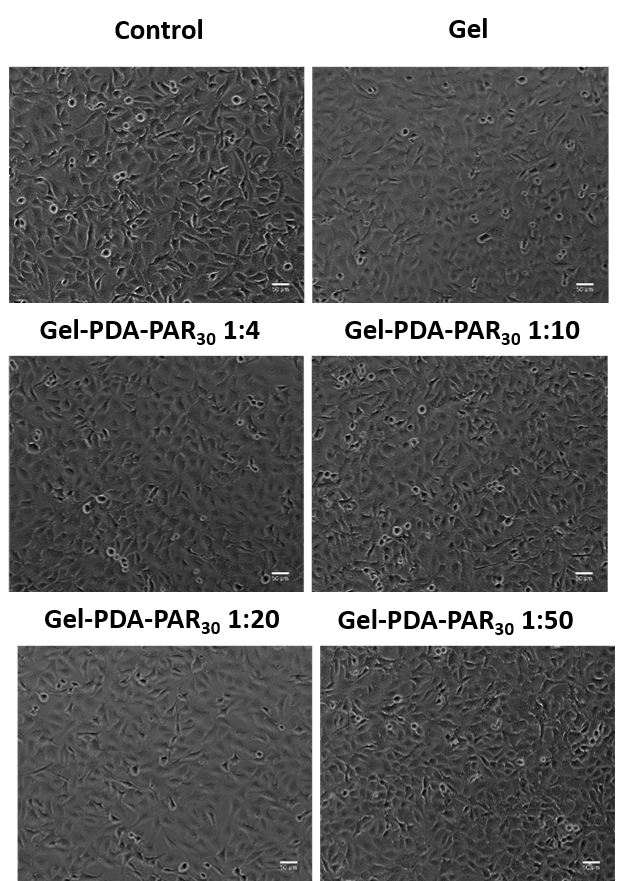


**Supplementary Figure 6**. Balb 3T3 cell morphology using bright field microscope after 24 hours of exposure with Gelatin NPs composite hydrogel loaded with NPs (PDAPAR_30_) at different dilutions (scale bar = 50µm).
